# Synthesis of Umbelliferone-Based, Thermally Stable, and Intrinsically Flame-Retardant Mono-Oxazine Benzoxazines: Understanding the Aminic Moiety’s Influence on Thermal Properties

**DOI:** 10.3390/polym17243340

**Published:** 2025-12-18

**Authors:** Trey Coughlin, Koki Weng, Maria Laura Salum, Pablo Froimowicz, Chris Scott, Hatsuo Ishida

**Affiliations:** 1 Department of Macromolecular Science and Engineering, Case Western Reserve University, Cleveland, OH 44106, USA; tcc48@case.edu (T.C.); kxs1006@case.edu (K.W.); mls323@case.edu (M.L.S.); pxf106@case.edu (P.F.); 2 Material Answers LLC, 66 Buckskin Drive, Weston, MA 02493, USA; cscott@materialanswers.com

**Keywords:** polybenzoxazine, umbelliferone, natural resource, renewable, thermal stability, flame retardant

## Abstract

A naturally sourced phenolic compound, umbelliferone, has been used to synthesize four monofunctional benzoxazines, two of which have been previously synthesized from aniline and furfurylamine. This study contributes two more—using benzylamine and phenethylamine—to provide insight into how the amine’s aromatic group and aliphatic chain length influence resulting properties. The proposed chemical structures of the novel monomers are confirmed by ^1^H nuclear magnetic resonance (^1^H-NMR) and ^1^H-^1^H nuclear Overhauser effect spectroscopy (NOESY). The polymerization behavior of each resin is determined by differential scanning calorimetry (DSC). The thermal degradation pattern and the flammability of each polymer are assessed by thermogravimetric analysis (TGA) and microscale combustion calorimetry (MCC), respectively. Char yields between 49% and 63% suggest the thermoset materials to be thermally stable and competitive for thermally demanding applications. All four polybenzoxazines demonstrate non-ignitable behavior, with heat release capacities below 100 J/g·K. Structure–property analyses on the two newly synthesized compounds have been provided to deepen our existing understanding of umbelliferone-benzoxazine systems, particularly regarding the effect of the aminic moiety on thermal properties.

## 1. Introduction

Polybenzoxazines are thermoset polymers obtained through the cationic ring-opening polymerization of 1,3-benzoxazines. Characterized by their six-membered oxazine ring fused with a benzene ring, a monomeric benzoxazine was first reported by Holly and Cope in 1944 [1]. Polybenzoxazines gained significant attraction in 1984, when the first bisphenol-A-based polybenzoxazine was patented [2,3,4]. However, despite a long history of monomer synthesis and industrial application patents, it was not until 1994 that the first detailed polybenzoxazine synthesis, structural analysis, and properties were reported [5]. Polybenzoxazines have since been recognized for outstanding properties, including near-zero shrinkage upon polymerization, low water absorption, remarkably high char yield, and a higher glass transition temperature than curing temperature. Benzoxazine resins also show low viscosity, making processing easier [6,7,8]. Desired for such properties, polybenzoxazines are now one of the very few polymers to have been newly commercialized within the past several decades. Applications for aerospace [9,10,11,12,13], coatings [14,15,16,17,18,19], electronics [20,21,22,23,24,25], and adhesives [26,27,28,29] are particularly attractive. Unique properties, such as very low surface-free energy without using fluorine atoms [30,31,32], anticorrosion [14,33,34,35], antimicrobial [36,37,38], antifouling [39,40,41], shape memory [42,43,44], self-healing [45,46], catalysis [47,48], and ambient or freeze drying of aerogel formation [49,50,51,52] are also noteworthy.

1,3-Benzoxazine monomers are obtained via the combination of a phenolic derivative, an aldehyde, and a primary amine in a one-pot Mannich condensation [8]. Formaldehyde is typically the aldehyde of choice, with alterations of the phenolic derivative or amine resulting in different properties of the resulting polymer; however, if desired, other forms of aldehyde, such as benzaldehyde [53,54], can replace formaldehyde. Solvent and solventless methods have both shown efficacy, often with high yields between 70 and 99 percent [8]. Despite the complexity of Mannich condensation, active groups of the starting compounds are typically retained [55]. Thus, the selection of the amine and phenolic derivative contributes greatly to the resulting polybenzoxazine’s properties. This avenue of modification drives much of polybenzoxazine’s high molecular design flexibility. Cationic ring-opening polymerization is achieved by heating the purified monomer at temperatures typically between 160 and 220 °C with or without added initiators and/or catalysts. Both monomers and higher oligomers could be present in benzoxazine resins, both of which can participate in the polymerization process. Benzoxazine polymerization is substantially less prone to the release of paraformaldehyde and amine byproducts than traditional phenolic curing [8].

Alongside growing interest in benzoxazines, the production and waste management of plastics has jumped to the forefront of environmental concern. Plastics have shown sharper growth than any other bulk material over the past few decades, with plastic production doubling between 2002 and 2023 [56]. Primary (non-recycled) plastic production alone contributed 2.24 gigatons of carbon dioxide equivalent in 2019, equating to 5.3% of global non-agricultural greenhouse gas emissions. Roughly 90% of these emissions have been shown to come from the production phase rather than the end-of-life (waste) phase of plastic life, though this is dwarfed in comparison to fossil-based fuel combustion [57]. Plastic production also presents sustainability issues: In 99% of cases, raw materials come from non-renewable sources, such as gas, coal, and, in the case of phenolic resins, petroleum [56]. Dwindling petroleum reserves, as well as the toxicity of bisphenol-A, have driven a search for phenolic replacements. Petroleum-based components may also require additional steps and energy to add functional groups [55] as opposed to natural compounds, which may already have desired functional groups incorporated into their structure. Since the prediction for the active investigation of natural-sourced benzoxazine research in 2011 [8], nearly one-third of benzoxazine papers now deal with bio-based benzoxazines.

Although research over the past decade has found viable natural replacements for feedstocks that could be used in plastic production, new plastics are rarely integrated into the chemical industry. The material properties of bio-based plastics are often inferior to their petroleum-based counterparts, which—on top of the time and cost put into scaling up—makes integration not worth the risk. Scalability is also limited by the availability of natural materials and the reproducibility of properties and quality [58]. It follows that, despite the existence of phenolic replacements and “greener” amines and solvents, biobased benzoxazines too have struggled to reach production at large scales. However, recent studies involving the incorporation of coumarin compounds show promise—coumarins are not only an abundant, naturally occurring replacement for nonrenewable phenolic compounds like bisphenol-A but the resulting polybenzoxazines also demonstrate thermal properties comparable to those of their petrochemical-based counterparts [19]. These compounds display low polymerization temperatures and high thermal stability once polymerized, and they have been synthesized in high yield [59]. Coumarins with a hydroxyl group in position 7, such as 7-hydroxycoumarin (umbelliferone) or 4-methylumbelliferone, are especially attractive for this application, as the unsubstituted ortho-position with respect to the oxygen atom in the heterocycle makes them conducive to the synthesis of 1,3-benzoxazines [60]. The unexpected high thermal stability of umbelliferone-based polybenzoxazine in comparison to petroleum-based raw materials, despite using natural-sourced raw material, was first recognized by Arza et al. in 2015 [61]. Strictly speaking, however, the incorporation of umbelliferone makes the resulting polybenzoxazine only *partially* bio-based; it would be desirable for both the phenolic and aminic moieties to be natural, renewable compounds. Consequently, researchers have attempted to synthesize polybenzoxazines using umbelliferone as the phenolic derivative and furfurylamine—a natural and renewable amine—in place of petroleum-based (and more typically used) aniline [60]. Notably, the polymerization temperature of the umbelliferone-furfurylamine benzoxazines was lower than that of other coumarin-containing resins. Thermal stability was exceptional, outperforming all other fully natural bio-based monofunctional benzoxazines and numerous petroleum-based benzoxazine resins [59].

The present work expands the umbelliferone-based benzoxazine repertoire by synthesizing two new, previously unreported umbelliferone-based benzoxazines. Their characterization and thermal properties are compared to those of two formerly reported umbelliferone-based benzoxazines, synthesized using furfurylamine and aniline. They are abbreviated as U-fa and U-a, respectively. The new compounds presented in this work—abbreviated as U-ba and U-pea—have been synthesized using benzylamine (ba) and phenethylamine (or 2-phenylethylamine) (pea), and they are identical to aniline except in one regard: the number of methylene groups between the aminic benzene ring and the nitrogen atom of the oxazine ring. Additionally, U-ba contains a phenyl group, where U-fa contains a furan group, revealing more about the activating effects in a comparative manner. Thus, in addition to gauging the thermal properties of new umbelliferone-based amines, this work aims to better understand how the aminic moiety contributes to the properties of an umbelliferone-containing polybenzoxazine.

## 2. Materials and Methods

### 2.1. Materials

Umbelliferone (>98%) was purchased from TCI Chemicals, Portland, OR, U.S.A., and used as received. Furfurylamine (C_5_H_7_NO) (>99%), aniline (C_6_H_7_N) (>99.5%), benzylamine (C_7_H_9_N) (99%), and phenethylamine (C_8_H_11_N) (99%) were used as received from Sigma-Aldrich, St. Louis, MO, USA. Paraformaldehyde (96%) was purchased from Acros Organics, Waltham, MA, USA, and used as received. Toluene (>99.5) was used as received from Fisher Chemical, Waltham, MA, USA, and chloroform (>99.8%) was used as received from Sigma-Aldrich. Sodium hydroxide pellets (98.8%) were purchased from Fisher Chemical, and basic alumina powder (particle size < 50 nm) was purchased from Sigma-Aldrich; both were used as received.

### 2.2. Synthesis of Umbelliferone-Containing Benzoxazines

Umbelliferone (0.970 g, 6 mmol), paraformaldehyde (0.400 g, 13.2 mmol), and 6 mmol of one of the four selected amines—furfurylamine (0.580 g), aniline (0.558 g), benzylamine (0.640 g), or phenethylamine (0.726 g)—were added to a 125 mL round-bottomed flask equipped with a magnetic stirring bar. To the dispersions containing furfurylamine, benzylamine, or phenethylamine, toluene (25 mL) was added, and the reaction mixture was refluxed with a condenser at 90 °C for 24 h. To the dispersion containing aniline, chloroform (25 mL) was added, followed by reflux at 80 °C for 24 h. In the case of U-a and U-ba, the crude product was passed through a P4 medium-porosity filter paper. The filtered U-a was diluted with 30 mL of chloroform and, in a 125 mL separatory funnel, washed three times with 1 M NaOH aqueous solution (15 mL each wash) and three times with distilled water (15 mL each wash). Crude U-fa and U-pea were passed through basic alumina powder under vacuum. For all samples, the solvent was evaporated, leaving behind crystals yellow in color. The U-fa crystals were recrystallized one time in toluene at low temperatures.

U-fa was obtained with a yield of 48%. ^1^H NMR (600 MHz, CDCl_3_, 20 °C) *δ*, ppm: 7.62 (1H, d, H_4c_, *J* = 9.4 Hz), 7.40 (1H, d, H_a_, *J* = 1.9 Hz), 7.26 (1H, d, H_7_, *J* = 8.5 Hz), 6.77 (1H, d, H_8_, *J* = 8.5 Hz), 6.33 (1H, t, H_b_, *J* = 3.3 Hz), 6.26 (1H, d, H_c_, *J* = 3.3 Hz), 6.24 (1H, d, H_3c_, *J* = 3.3 Hz), 4.94 (2H, s, H_2_, *J* = 9.4 Hz), 4.21 (2H, s, H_4_), 3.90 (2H, s, H_d_). ^13^C NMR (126 MHz, CDCl_3_) *δ*, ppm: 161.13, 157.57, 152.69, 151.03, 144.03, 142.87, 126.80, 113.83, 112.86, 112.40, 110.48, 109.41, 107.85, 82.50, 48.74, 44.71.

U-a was obtained with a yield of 68%. ^1^H NMR (600 MHz, CDCl_3_, 20 °C) *δ*, ppm: 7.95 (1H, d, H_4c_, *J* = 9.5), 7.47 (1H, d, H_7_, *J* = 8.6), 7.25 (2H, t, H_b_, *J* = 8.7), 7.17 (2H, t, H_c_, *J* = 1.5), 6.90 (1H, t, H_a_, *J* = 9.5), 6.79 (1H, d, H_8_, *J* = 8.5), 6.29 (1H, d, H_3c_, *J* = 9.5), 5.56 (2H, s, H_2_), 4.76 (2H, s, H_4_). ^13^C NMR (126 MHz, CDCl_3_) *δ*, ppm: 161.10, 157.86, 151.87, 147.99, 144.07, 129.53, 129.18, 128.37, 126.99, 122.20, 118.65, 114.17, 112.91, 112.35, 109.02, 79.99, 46.30.

U-ba was obtained with a yield of 59%. ^1^H NMR (600 MHz, CDCl_3_, 20 °C) *δ*, ppm: 7.97 (1H, d, H_4c_, J = 9.5 Hz), 7.51 (1H, d, H_7_, *J* = 8.6 Hz), 7.34 (2H, t, H_b_, *J* = 6.0 Hz), 7.33 (2H, t, H_c_, *J* = 6.0 Hz), 7.32 (1H, d, H_a_), 6.84 (1H, d, H_8_, *J* = 8.5 Hz), 6.26 (1H, d, H_3c_, *J* = 9.5 Hz), 4.99 (2H, s, H_2_), 4.03 (2H, s, H_4_), 3.89 (2H, s, H_d_). ^13^C NMR (126 MHz, DMSO) *δ*, ppm: 160.02, 157.19, 152.11, 144.77, 137.95, 128.54, 128.42, 127.34, 127.14, 113.28, 111.99, 111.97, 107.39, 82.61, 54.88, 43.47.

U-pea was obtained with a yield of 69%. ^1^H NMR (600 MHz, CDCl_3_, 20 °C) *δ*, ppm: 7.54 (1H, d, H_4c_, J = 9.5 Hz), 7.20 (1H, t, H_b_, *J* = 8 Hz), 7.16 (1H, d, H_7_, *J* = 8.6 Hz), 7.12 (2H, t, H_c_, *J* = 7.4 Hz), 7.11 (1H, t, H_s_), 6.66 (1H, t, H_8_, *J* = 9.5 Hz), 6.16 (1H, d, H_3c_, *J* = 9.5 Hz), 4.87 (2H, s, H_2_), 4.17 (2H, s, H_4_), 2.94 (2H, t, H_e_, *J* = 7.3 Hz), 2.82 (2H, t, H_d_, *J* = 7.1 Hz). ^13^C NMR (126 MHz, CDCl3) *δ*, ppm: 161.24, 157.92, 152.65, 144.12, 139.54, 128.80, 128.56, 126.71, 126.40, 113.80, 112.69, 112.22, 108.28, 83.56, 53.69, 45.10, 34.91.

### 2.3. Polymerization Procedure

The onset and peak polymerization temperatures for each benzoxazine monomer were determined according to its DSC thermogram. Benzoxazine monomers were isothermally heated at their melting endsets for 1 h to ensure complete melting. They then underwent 3 h of isothermal heating at the onset polymerization temperature, 1 h at the peak polymerization temperature, and 2 h at the polymerization endset temperature. Thus, U-fa was heated at 180, 187, 197, and 206 °C; U-a at 146, 178, 194, and 221 °C; U-ba at 125, 210, 221, and 235 °C; and U-pea at 130, 200, 213, and 222 °C (see Table 1). Heating was achieved using a Being BON-30T 30L (Novatech International Inc., Kingwood, TX, USA) natural convection oven under standard atmospheric composition and pressure.

### 2.4. Characterization

Proton nuclear magnetic resonance (^1^H NMR) spectra were obtained through the use of a Bruker Avance spectrometer at a proton frequency of 500 MHz and a relaxation time of 1 s (Bruker Corp., Billerica, MA, USA) Differential scanning calorimetric (DSC) thermograms were obtained by TA Instruments (New Castle, DE, USA) model DSC Q2000 with a nitrogen flow rate of 60 mL/min. Thermograms were recorded using a heating rate of 10 °C/min and a sample mass of 1–3 mg held in an aluminum pan. Thermogravimetric analysis (TGA) was carried out using TA Instruments model TGA 500 to gauge the thermal decomposition of polybenzoxazines. For each polybenzoxazine, a single run was performed with a sample weight of 2–5 mg from room temperature to 850 °C at a heating rate of 10 °C/min and a nitrogen flow rate of 60 mL/min. Data was collected up to 800 °C, and samples were held in platinum pans. Microscale combustion calorimetry (MCC) data were acquired using a Deatak (Chicago, IL, USA) model MCC-4 and around 2 mg of each polymerized sample. An oxygen flow rate of 20 mL/min with a heating rate of 1 °C/s from 75 to 800 °C was used.

## 3. Results and Discussion

The current understanding of umbelliferone-containing benzoxazines is limited to the umbelliferone moiety and benzoxazine nucleus. It is understood that the C=C double bond at position *3c* of the umbelliferone moiety, conjugated with the electron-drawing carbonyl group of the ester, imparts an activating effect on the monomer by behaving as a Michael acceptor. The activating effect manifests as lower polymerization temperatures, slower thermal degradation, and a greater degree of crosslinking when compared to benzoxazines lacking such a substituent, such as phenol and aniline-based mono-oxazine benzoxazine (PH-a) [59]. Additional substituents that weaken this activating effect—for instance, a methyl group at position *4* (seen in 4-methylumbelliferone) [60]—result in a higher polymerization temperature and steeper degradation profile but not necessarily a lower glass transition temperature, as the steric effects of the additional substituent also influence chain mobility.

**Scheme 1:**
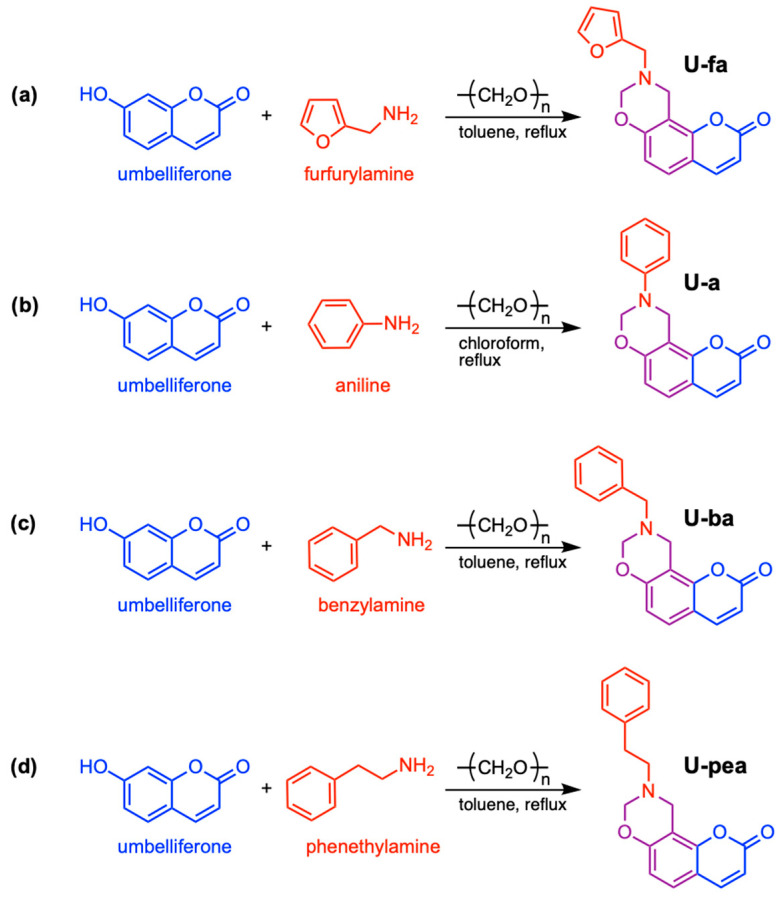
Reaction diagram towards the synthesis of (**a**) U-fa, (**b**) U-a, (**c**) U-ba, (**d**) and U-pea.

The relationship between the amine and the resulting properties of the umbelliferone-containing benzoxazine is not as thoroughly understood. Two different amines—aniline and furfurylamine—have been previously used in the synthesis of umbelliferone-containing benzoxazines (U-a and U-fa, respectively), but they have not been compared [59,61]. Regarding U-fa, it has been generally understood that the furfuryl group has an activating effect on polymerization [59]. This behavior likely stems from the ability of furan’s oxygen heteroatom to participate in H-bonding, as well as the donation of electron density from this oxygen to its adjacent carbon atoms in the ring, making them susceptible to electrophilic aromatic substitution (and thus crosslinking) [62]. However, U-fa and U-a differ in aliphatic chain length and aromatic group (furan versus benzene), making a direct comparison between the effects of their aromatic groups challenging. This study bridges that gap by synthesizing two more umbelliferone-containing benzoxazines: umbelliferone-benzylamine benzoxazine (U-ba) and umbelliferone-phenethylamine benzoxazine (U-pea). The synthesis of these two new compounds is presented in Scheme 1, in addition to the U-a and U-fa resins prepared for this study. The selection of amines used for this study allows for a more complete comparison via the following structural parameters:

- U-a, U-ba, and U-pea differ only in the number of aliphatic carbons between the aminic benzene ring and the benzoxazine nucleus.
- U-a contains no aliphatic carbons in its amine group.
- U-ba contains one aliphatic carbon.
- U-pea contains two aliphatic carbons.
- U-fa, rather than containing a benzene ring separated from the oxazine ring by one aliphatic carbon, contains a furan ring separated by one aliphatic carbon.
- Because of this last point, U-fa can be compared most immediately to U-ba, providing insight into the activating effect of the furfuryl group.

### 3.1. Nuclear Magnetic Resonance and Nuclear Overhauser Effect Spectroscopy of the Monomers

**Figure 1:**
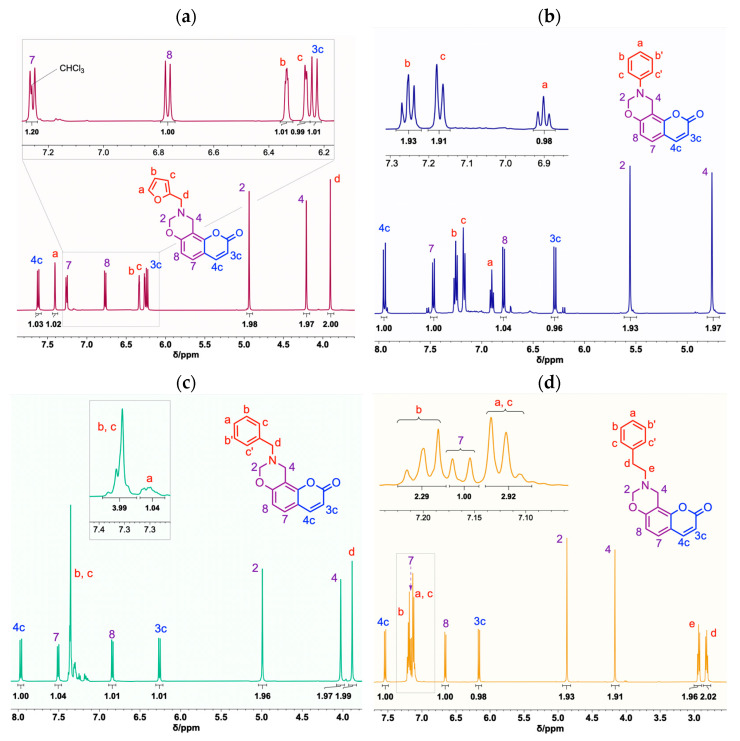
^1^H-NMR spectra of (**a**) U-fa, (**b**) U-a, (**c**) U-ba, and (**d**) U-pea. Peak integrations have been provided beneath the baseline.

**Figure 2:**
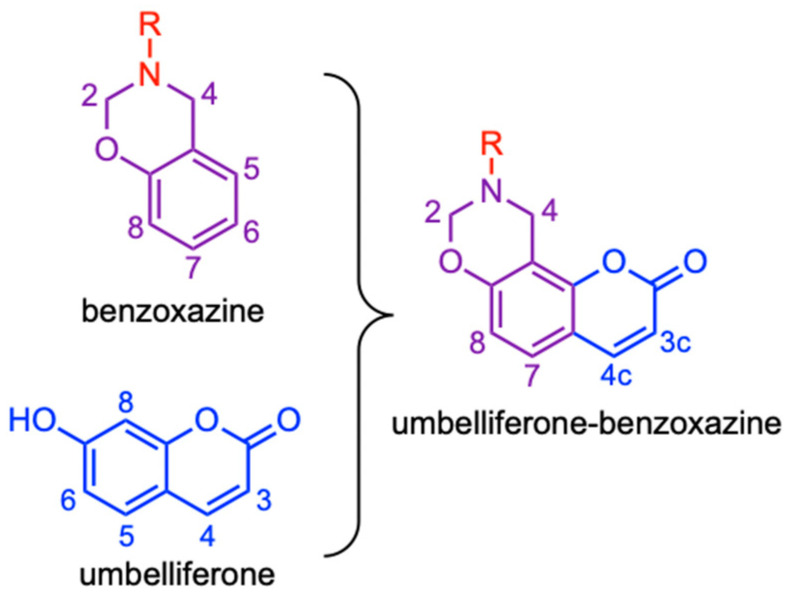
Labelling scheme of umbelliferone-based benzoxazine and the compounds it is derived from.

Before assessing the properties of each resin and resulting polymer, the monomer structures are verified using nuclear magnetic resonance (NMR) spectroscopy. The ^1^H NMR spectra for U-a, U-fa, U-ba, and U-pea are shown in Figure 1. Like previous works studying coumarin-containing benzoxazines, this work will view the synthesized compounds as benzoxazine derivatives rather than umbelliferone derivatives, despite their shared benzene ring. Thus, the numberings shown on each spectrum follow the numbering shown in Figure 2. The benzoxazine nucleus is numbered according to established benzoxazine labeling conventions, while the positions on the strictly umbelliferone component are labeled with an additional “*c*” for “coumarin”. Aminic components will be labeled using lowercase letters.

**Figure 3:**
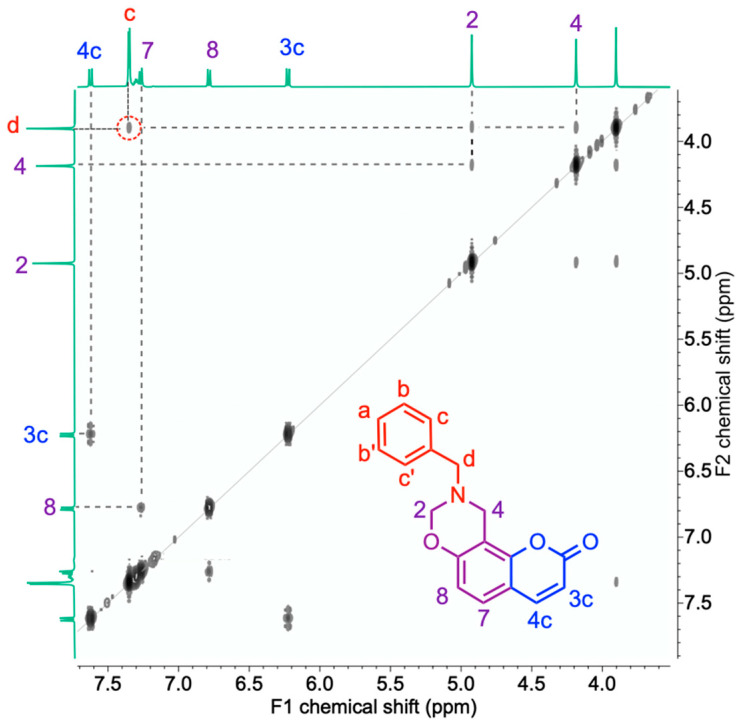
^1^H-^1^H nuclear Overhauser effect spectrum for U-ba. The signal indicating interaction between H_c_ and H_d_ is circled in red.

Two characteristic benzoxazine singlets, attributed to the methylene groups on either side of the heteroatom in the oxazine ring (their positions labeled 2 and 4), occur around 4.0 and 5.0 ppm for all four compounds and integrate at a 1:1 ratio to one another. The frequency separation of the majority of benzoxazines of these singlets is in the range of 0.7–0.9 ppm with only a few exceptions [8]. In the case of U-pea and U-a, these are the only singlets in the lower field of the spectrum, making their assignments relatively straightforward. U-ba, however, contains a third methylene group (H_d_) with an electronic environment comparable to that of H_4_. Accordingly, the spectrum for U-ba displays an additional singlet close to 4.0 ppm, complicating the assignment of the methylene groups at positions *4* and *a*. U-fa’s structure, which also contains a third methylene group on the nitrogen heteroatom, has been previously elucidated using nuclear Overhauser effect spectroscopy (NOESY) [59]. The same approach can be carried out for U-ba. The NOESY spectrum for U-ba, shown in Figure 3, displays through-space interaction (circled in red) between H_c_ and the proton generating the 3.86 ppm signal, but not between H_b_ and the proton generating the 4.19 ppm signal. According to the proposed structure, H_c_ would have stronger interaction with H_d_ than it would with H_4_, as H_d_ lies in closer proximity. Following this rationale, the peak at 3.86 ppm corresponds to H_d_, confirming the peak assignments shown in Figure 1c.

Common to all umbelliferone-containing benzoxazines, four doublets situated between 6.0 and 8.0 ppm result from the single protons on the umbelliferone moiety and the benzene ring of the benzoxazine nucleus. These doublets, which integrate at half that of the methylene groups in positions *2* and *4*, arise from two pairs of interacting single protons on adjacent carbon atoms. Proton *4c* is heavily deshielded due to a mesomeric effect with the carbonyl group, resulting in its larger chemical shift. Proton *3c* is positioned in the *α* position rather than the *β* position with respect to the carbonyl group, giving rise to a smaller chemical shift. Of the two remaining doublets, proton *7* is conjugated with the carbonyl, but—unlike proton *4c*—it does not occupy the *β* position, resulting in a chemical shift greater than proton *8* but less than proton *4c.* Protons *7* and *8* participate in an aromatic system, generating a greater chemical shift than proton *3c*. Had the oxazine ring closed on the other ortho-position with respect to umbelliferone’s hydroxyl group, the product would instead contain two isolated protons in the benzoxazine nucleus, generating two additional singlets in the ^1^H NMR spectrum. This holds true for both U-ba and U-pea. While the peak couplings are not readily apparent from the ^1^H NMR spectra, a cursory look at the ^1^H-^1^H NOESY data for U-ba in Figure 3 confirms that proton *4c* interacts with proton *3c*, and proton *7* interacts with proton *8,* with no crossover. This supports that protons *7* and *8* are, in fact, in neighboring positions on the benzene ring.

Signals arising from the aromatic aminic moiety occur between 6.0 and 7.5 ppm. The spectra for U-ba and U-pea show overlapping peaks and are thus labelled in groups. Due to the symmetry of the benzene ring in U-a, U-ba, and U-pea, peaks H_b_ and H_c_ appear to integrate at a ratio twice that of H_a_.

### 3.2. Dynamic Scanning Calorimetry of the Monomers

**Figure 4:**
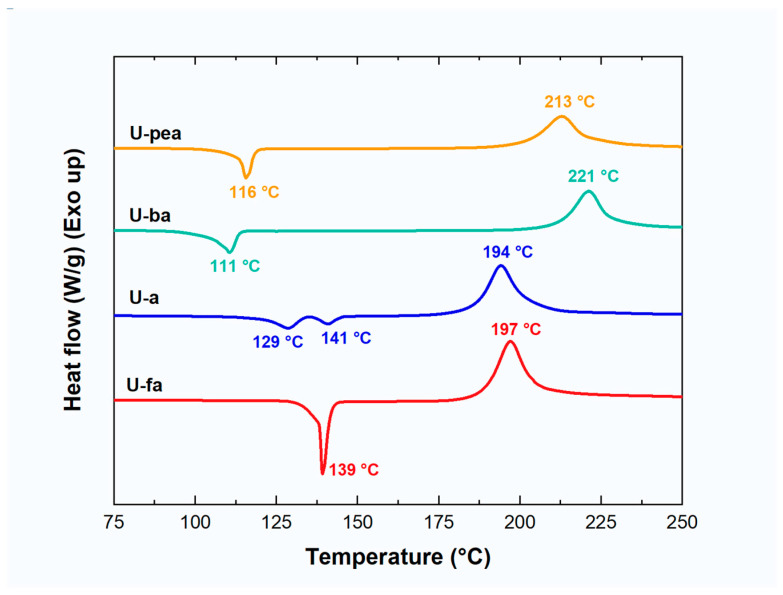
DSC thermograms of the four umbelliferone-based benzoxazine monomers. Peak melting and peak polymerization temperatures have been included.

The DSC thermogram for each monomer at a heating rate of 10 °C/min is included in Figure 4. Each resin shows an endotherm, attributed to the melting of the resin’s crystals, and an exotherm, attributed to the resin’s polymerization. At a heating rate of 10 °C/min, peak melting temperatures occurred at 111 °C for U-ba, 116 °C for U-pea, 129 and 141 °C for U-a, and 139 °C for U-fa, with the sharpness of U-fa’s melting peak suggesting high purity. The two distinct melting peaks of U-a are suspected to arise from the presence of more than one type of crystal. Peak polymerization temperatures occurred in the following (ascending) order: U-a at 194 °C, U-fa at 197 °C, U-pea at 213 °C, and U-ba at 221 °C.

Although the polymerization temperatures of U-ba and U-pea were higher than those seen for U-a and U-fa, they are still low enough to suggest that the activating effect of the umbelliferone moiety is preserved (for comparison, PH-a’s polymerization usually occurs around 265 °C [58]). Unexpectedly, the peak polymerization pattern deviates from the order of amine aliphatic chain length: The compound with the greatest aliphatic chain length—U-pea—has a polymerization temperature below that of U-ba but above U-a, which has the smallest aliphatic chain length. While this does mean U-ba and U-pea require higher temperatures to polymerize, it also gives them a much larger processing window than U-a and U-fa, considering that U-ba and U-pea’s peak melting temperatures are also lower.

**Figure 5:**
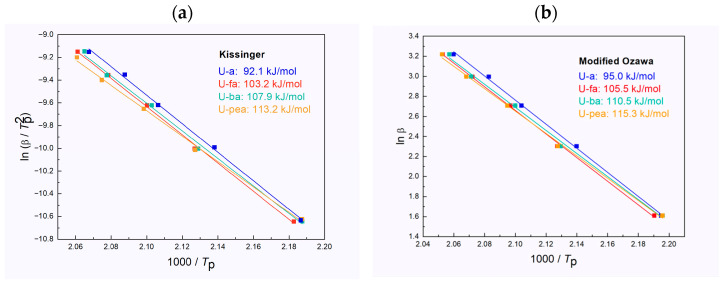
(**a**) Kissinger and (**b**) modified Ozawa plots for the four umbelliferone-benzoxazine monomers. For each monomer, the activation energy toward polymerization is provided.

The peak exotherm temperature across five different DSC heating rates (5, 10, 15, 20, and 25 °C/min) was compiled and plotted according to the Kissinger and modified Ozawa equations, shown below, to determine the activation energy of polymerization for each monomer:

(1) $$\ln\left(\frac{\beta }{{T_{p}}^{2}}\right)\;=\;\ln\left(\frac{AR}{E_{a}}\right)\;-\;\frac{E_{a}}{RT_{p}}\;\mathrm{Kissinger}\;\mathrm{Eq}.$$

(2) $$\;\ln\beta =-1.052\frac{E_{a}}{RT_{p}}+C\;\;\mathrm{Modified}\;\mathrm{Ozawa}\;\mathrm{Eq}.$$

where *β* is the constant heating rate, *T*_p_ is the peak polymerization temperature, *E*_a_ is the activation energy for polymerization, *R* is the gas constant, and *A* is the frequency factor. The plots of each monomer according to each method and the corresponding activation energies toward polymerization are shown in Figure 5. It can be observed that the monomers containing a longer aminic aliphatic chain demonstrate higher activation energies. U-fa, despite having the same aliphatic chain length as U-ba, exhibits lower activation energy, suggesting a greater activating effect of the furan ring over U-ba’s benzene ring. U-a, having no aliphatic chain, exhibits the lowest activation energy towards polymerization of the four monomers. This pattern may be driven by the greater steric hindrance induced by the bulkier amine groups: As the aliphatic chain length of the aminic portion increases, the monomer itself becomes bulkier, and the steric effects of these increasingly bulky groups have an antagonistic effect on polymerization, effectively raising the activation energy. In addition to the increased side group size, the rotation around the N-C bond of the aminic group occupies a greater radius than the simple rotation of the benzene ring around the N-C bond, further increasing the steric hindrance of the aminic groups other than aniline.

### 3.3. Thermogravimetric Analysis of the Polymers

**Figure 6:**
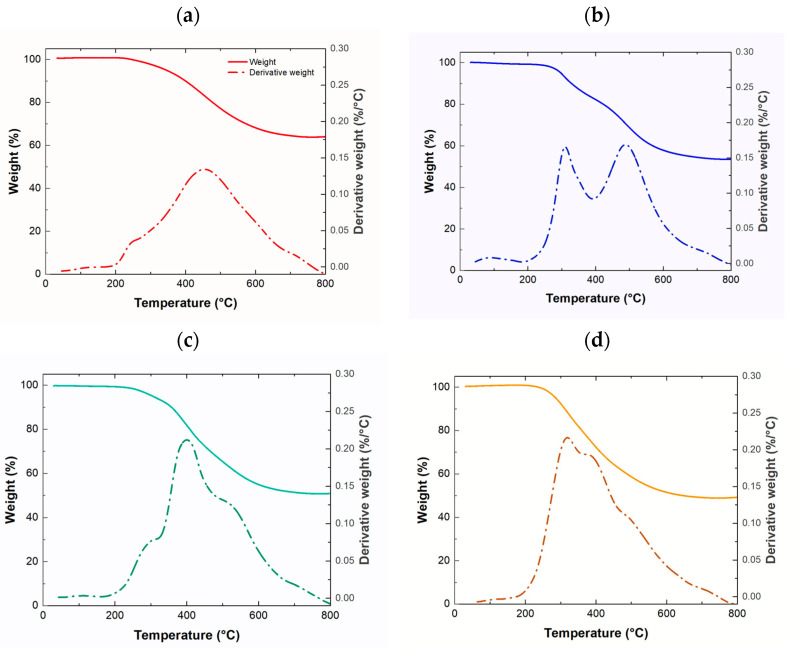
TGA thermogram for (**a**) poly(U-fa), (**b**) poly(U-a), (**c**) poly(U-ba), and (**d**) poly(U-pea). Both residual weight (—) and derivative weight (---) are shown.

Thermogravimetric analysis (TGA) was run to understand the thermal decomposition pattern of each polymer. The thermograms are shown in Figure 6, with derivative weight included to more clearly show each degradation event. Polybenzoxazines typically follow a three-step decomposition pattern: evaporation of the amine at chain ends, amine evaporation from the main chain, and breakage of the phenolic linkage coincident with degradation of the Mannich base [63,64]. The derivative weight thermogram for poly(U-a) shows two thermal events, with the first peaking around 320 °C, attributed to evaporation of aniline at the terminal groups. The second thermal event, centered around 500 °C, likely corresponds to aniline evaporation from the main chain. Poly(U-fa) shows one broad peak centered around 475 °C, with a small shoulder at 250 °C. With three distinct peaks, Poly(U-ba) appears to most closely resemble the traditional decomposition pattern—its peaks occur at 275, 510, and 550 °C, with the fastest rate of degradation occurring at 510 °C. Poly(U-pea) shares a similar scheme, with its sharpest degradation peak occurring at 275 °C.

In addition to comparing *T*_d5_ and *T*_d10_ values, the char yield of each compound has been related to the limiting oxygen index (LOI) using the van Krevelen equation, as shown below [65]:

(3) LOI = 17.5 + 0.4Yc

where Y*c* is the char yield of the polymer. Poly(U-fa) had the highest LOI of 43, followed by poly(U-a) at 39, poly(U-ba) at 38, and poly(U-pea) at 37. LOI quantifies the minimum oxygen content required to sustain combustion of a material, with an LOI above 28 considered non-ignitable for polymers [66]. Thus, based on the predictions of their LOI value, all four polymers may be deemed non-ignitable. Other predictors of flammability behavior, such as heat release capacity and total heat release, can be obtained via microscale combustion calorimetry, as explored below.

*T*_d5_ and *T*_d10_, defined as the temperatures at which degradation is 5% and 10%, respectively, and char yield (here defined as the residual weight at 800 °C) can be used to quantitatively compare the thermal stabilities of the four polymers. These values are compiled in Table 2. Poly(U-fa) showed the highest char yield at 64%, followed by poly(U-a) (52.5%), poly(U-ba) (50%), and poly(U-pea) (49%). For reference, poly(PH-a)—a non-renewable polybenzoxazine synthesized from phenol and aniline—typically has a char yield around 40%.

The significantly higher char yield of poly(U-fa) can be rationalized by the activating effect of its furan group, which itself is able to participate in polymerization due to its oxygen heteroatom and C=C group. The furan ring’s participation in crosslinking results in the generation of fewer volatile fractions, whereas the other three compounds contain a phenyl group bound to the polymer network by an aliphatic carbon chain only. Thus, the latter is more easily evaporated from the polymer, leaving behind less material by 800 °C.

Excluding poly(U-fa), the compounds with longer aliphatic chain lengths exhibited lower char yields. As the number of carbon-carbon bonds between the oxazine ring and aromatic ring of the amine increases, so does the number of bonds, which, if broken, would generate a volatile fraction (and likely trigger the degradation of the rest of the aliphatic chain). However, even if we consider a scenario where poly(U-fa), poly(U-ba), and poly(U-pea) all had the C-C bond nearest the oxazine ring broken, the pattern will persist. This is more an artifact of residual weight being measured with respect to the initial sample weight: The aminic moiety of U-pea is greater than that of U-ba, which is greater than that of U-a, so the volatilization of the entire aminic moiety will be reflected as a greater reduction in weight following the same pattern. Thus, looking at other values may be more suitable for comparing the degradation of these three compounds.

Using both *T*_d5_ (5% decomposition temperature) and *T*_d10_ (10% decomposition temperature) as references, the polymer that retained the most residual weight in its early stages of degradation was poly(U-fa), followed by poly(U-ba), poly(U-a), and poly(U-pea), in this order. Interestingly, the pattern seen here deviates from the order of aliphatic chain length, with poly(U-ba) reaching higher temperatures than either of the other two non-furan-containing polybenzoxazines before reaching 90% residual weight. It is possible that an anilinic benzene ring immediately adjacent to the amine group may be less reactive than a benzene group that is screened by a CH_2_ group between the amine and benzene ring. Although these benzene rings do not show high reactivity, they, nonetheless, partially react to participate in the crosslinking networks; the degree of participation in the case of poly(U-ba) may indeed be greater than that of poly(U-a).

### 3.4. Microscale Combustion Calorimetry of the Polymers

Microscale combustion calorimetry (MCC) creates an environment conducive to the combustion of just milligrams of samples and measures the heat release rate (HRR), or heat emitted in a fire, across a temperature range. From this, the total heat release (THR), represented by the area under the HRR curve, can be measured. The heat release capacity (HRC) is considered a good quantitative parameter for flammability and the ignition behavior of polymers [67,68,69]. HRC is calculated based on the maximum specific HRR (accounting for the initial sample mass) and the experimental heating rate. An HRC below 300 J/g·K is considered to be self-extinguishing, while those under 100 J/g·K are considered nonignitable [66]. HRC was used to study the flammability characteristics of polybenzoxazines [69,70,71,72,73,74]. The majority of polybenzoxazines studied resulted in non-combustible ratings. Table 3 compiles the HRC and THR values for each of the four umbelliferone-based polybenzoxazines.

Poly(U-fa) yielded the lowest HRC of 27 J/g·K followed by poly(U-a) at 46 J/g·K, poly(U-ba) at 72 J/g·K, and poly(U-pea) at 78 J/g·K. Thus, all four compounds can be deemed nonignitable, making them highly promising for fire-retardant applications. It is important to recognize that they are intrinsic, non-ignitable polymers without any added flame retardants. The sequence seen here mirrors that of descending char-yield, with poly(U-fa) seeing the highest char-yield and poly(U-pea) the lowest. Char formation is one factor that decreases a polymer’s flammability—a more substantial char layer insulates the underlying polymer from heat and prevents the escape of evaporated fuels, resulting in a lower HRC. The significantly low HRC value for poly(U-fa) lies once again in the activating effect of the furan group, which participates in the polymer’s crosslinking. More energy needs to be put into a more densely crosslinked network to produce the same heat release as a more sparsely crosslinked network. The C-C bonds in these linear chains are also relatively weak, making them easier to break, releasing more volatile components.

## 4. Conclusions

Two novel coumarin-containing benzoxazines, U-ba and U-pea, were successfully synthesized using 7-hydroxycoumarin (umbelliferone) as a natural and renewable phenol source. Their thermal properties were studied and compared to previously reported coumarin-containing benzoxazines, U-a and U-fa. The novel resins showed polymerization temperatures higher than both U-a and U-fa, but within a comparable range, indicating that the activation effect of the umbelliferone moiety was preserved. Comparing the activation energies of the four monomers towards polymerization revealed that a smaller aliphatic chain length lowered the activation energy and that the furfuryl group in U-fa had a significant activating effect when compared to U-ba’s benzyl group. The novel umbelliferone-containing polybenzoxazines exhibited good thermal stability and low flammability, as evidenced by char yields above 49% and heat release capacities well below 100 J/g·K. Longer aliphatic chain lengths were associated with lower char yields and higher heat release capacities, with poly(U-fa) yielding an unexpectedly low heat release capacity of 27 J/g·K. Poly(U-ba) showed the highest *T*_d5_ and *T*_d10_ values (305 and 358 °C) among the three non-furfuryl containing polymers. The findings of this study reinforce the promising thermal properties of existing umbelliferone-based benzoxazines and expand their breadth to include the newly synthesized U-ba and U-pea benzoxazines. A deeper understanding of the aminic moiety’s influence in umbelliferone-benzoxazine systems allows for a more tailored and intentional approach when designing new coumarin-based benzoxazines in the future, particularly for thermally demanding applications.

## Figures and Tables

**Table 1:** Melting peak and endset temperatures and polymerization onset, peak, and endset temperatures for each monomer at a heating rate of 10 °C/min.

Monomer	Melting Peak (°C)	Melting Endset (°C)	Polymerization Onset (°C)	Polymerization Peak (°C)	Polymerization Endset (°C)
U-fa	139	180	187	197	206
U-a	129, 141	133, 146	178	194	221
U-ba	111	125	210	221	235
U-pea	116	130	200	213	222

**Table 2:** Char yield (%) for each polymer, taken as the percent of residual sample at 800 °C, and *T*_d5_ and *T*_d10_, taken as the temperature at which 5 and 10% degradation occurs. Limiting oxygen index (%), calculated from char yield, has been included.

Polymer	Char Yield (%)	Limiting Oxygen Index (LOI)	*T*_d5_ (°C)	*T*_d10_ (°C)
Poly(U-fa)	64	43	346	400
Poly(U-a)	53	39	292	325
Poly(U-ba)	51	38	305	358
Poly(U-pea)	49	37	286	312

**Table 3:** Heat release capacity (J/g·K) and total heat release (kJ/g) for each polymer, according to microscale combustion calorimetry data.

Polymer	Heat Release Capacity (HRC) (J/g·K)	Total Heat Release (THR) (kJ/g)
Poly(U-fa)	27	5.3
Poly(U-a)	46	7.9
Poly(U-ba)	72	10.1
Poly(U-pea)	78	12.8

## Data Availability

The raw data supporting the conclusions of this article will be made available by the authors upon request.
